# Evaluation of the reinforced integrated disease surveillance and response strategy using short message service data transmission in two southern regions of Madagascar, 2014–15

**DOI:** 10.1186/s12913-018-3081-2

**Published:** 2018-04-10

**Authors:** Rado Randriamiarana, Grégoire Raminosoa, Nikaria Vonjitsara, Rivo Randrianasolo, Harena Rasamoelina, Harimahefa Razafimandimby, Arthur Lamina Rakotonjanabelo, Richard Lepec, Loïc Flachet, Ariane Halm

**Affiliations:** 1 Indian Ocean Field Epidemiology Training Programme, SEGA One Health Network, Indian Ocean Commission, Ebène, Mauritius; 2 Epidemiological Surveillance Department, Ministry of Health, Antananarivo, Madagascar; 3 Health Surveillance Unit, SEGA One Health Network, Indian Ocean Commission, Ebène, Mauritius; 4 Surveillance Unit, World Health Organisation, Antananarivo, Madagascar

**Keywords:** Integrated disease surveillance and response IDSR, Madagascar, Basic healthcare, Infectious diseases, Surveillance

## Abstract

**Background:**

The Integrated Disease Surveillance and Response (IDSR) strategy was introduced in Madagascar in 2007. Information was collected by Healthcare structures (HS) on paper forms and transferred to the central level by post or email. Completeness of data reporting was around 20% in 2009–10. From 2011, in two southern regions data were transmitted through short messages service using one telephone provider. We evaluated the system in 2014–15 to determine its performance before changing or expanding it.

**Methods:**

We randomly selected 80 HS and interviewed their representatives face-to-face (42) or by telephone (38). We evaluated knowledge of surveillance activities and selected case definitions, number of SMS with erroneous or missing information among the last ten transferred SMS, proportion of weekly reports received in the last 4 weeks and of the last four health alerts notified within 48 h, as well as mobile phone network coverage.

**Results:**

Sixty-four percent of 80 interviewed HS representatives didn’t know their terms of reference, 83% were familiar with the malaria case definition and 32% with that of dengue. Ninety percent (37/41) of visited HS had five or more errors and 47% had missing data in the last ten SMS they transferred. The average time needed for weekly IDSR data compilation was 24 min in the Southern and 47 in the South-eastern region. Of 320 expected SMS 232 (73%) were received, 136 (43%) of them in time. Out of 38 alerts detected, four were notified on time. Nine percent (7/80) of HS had no telephone network with the current provider.

**Conclusions:**

SMS transfer has improved IDSR data completeness, but timeliness and data quality remain a problem. Healthcare staff needs training on guidelines and case definitions. From 2016, data are collected and managed electronically to reduce errors and improve the system’s performance.

## Background

Early detection of epidemics, but also population health status ascertainment and Public Health decision-making oftentimes depend on effective disease surveillance systems [1, 2].

The Integrated Disease Surveillance and Response (IDSR) approach was proposed to countries in the African region by the Regional Office for Africa of the World Health Organization (WHO AFRO) in 1998. Its purpose is to establish one national communicable disease surveillance system integrating different surveillance activities into one, consisting of functions using the same or similar structures, processes and personnel. The goal is an effective communicable disease control based on functioning effective disease surveillance and response systems. Since then, many countries in Africa have adapted and adopted it with some including non-communicable diseases as well [3, 4].

The International Health Regulations (IHR) constitute an agreement by WHO member states to (implement measures limiting the spread of health risks, including requirements concerning surveillance and response activities [5]. IDSR systems can help achieve and sustain countries’ IHR obligations, as priorities like timely detection and response to Public Health (PH) events are shared between the two [6].

In Madagascar, the IDSR strategy was adapted to the national context with help of the WHO and introduced in 2007. Healthcare Structures (HS) collected information on paper forms and transferred it to the central level by post or email. The mean national completeness of data reporting in 2011–13 was 20%. The system was non-representative and not responding to its objectives (source: Direction de Veille Sanitaire et Surveillance Epidémiologique, DVSSE, 2010).

**Fig. 1:**
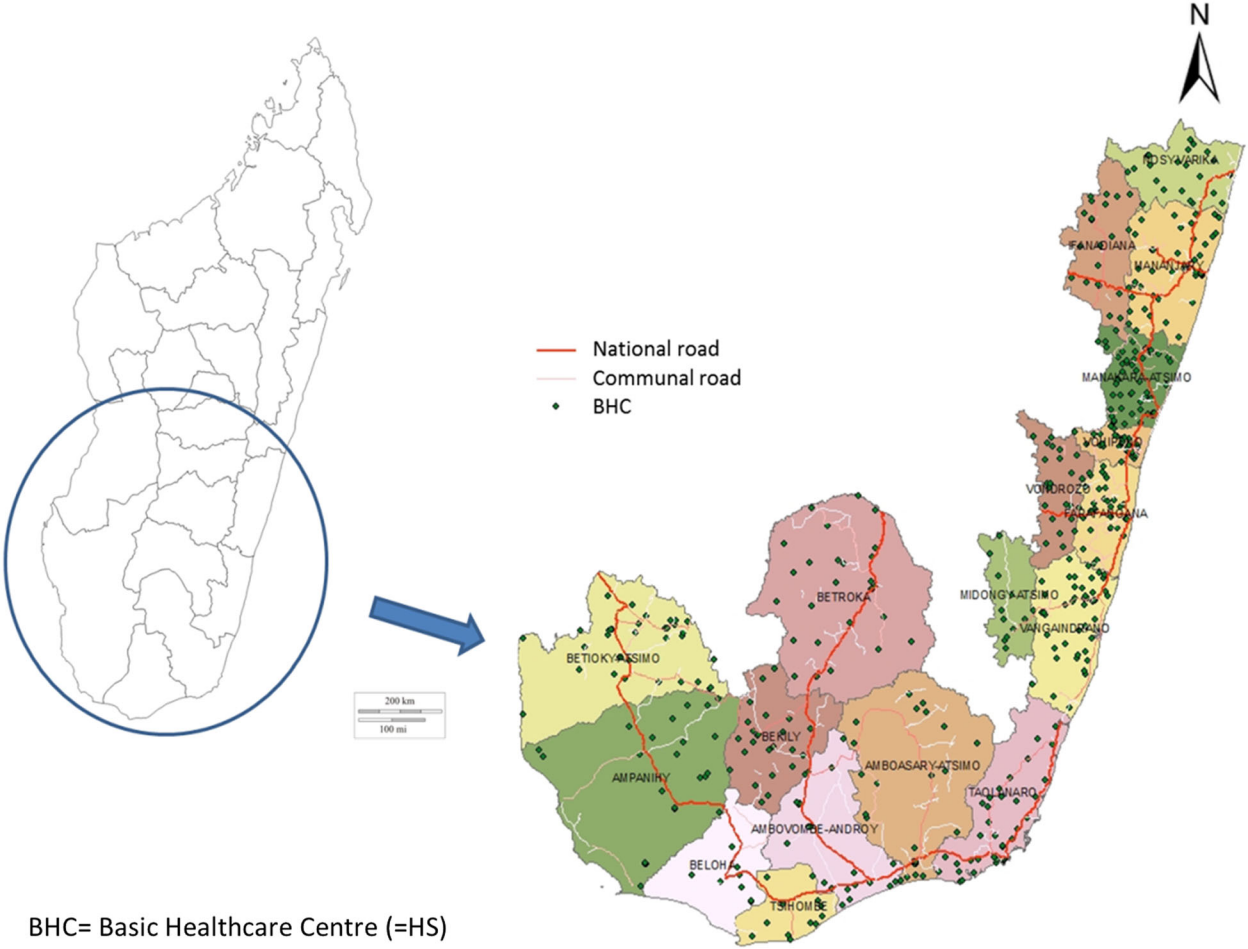
Madagascar’s 18 south and southeast districts targeted by the reinforced IDSR strategy, 2013. Direction de Veille Sanitaire et Surveillance Epidémiologique, Madagascar. Map of Madagascar and its southern regions and districts pointing out the main road network and HS locations

The south and southeast of Madagascar consists of five regions with 18 districts that are vulnerable to epidemic threats through their regular and alternating droughts and inondations leading to nutrition crises, locusts plagues, and a general unfavourable socio-economic context (Fig. 1: Madagascar’s 18 south and southeast districts targeted by the reinforced IDSR strategy, 2013, source: DVSSE).

From 2011, the Central Emergency Response Fund (CERF) and WHO, in collaboration with the Ministry of Health through the Direction de Veille Sanitaire et Surveillance Epidémiologique (DVSSE) started reinforcing the IDSR strategy in the three regions in the south. Of their 238 HS, 152 (64%) were covered by the selected mobile phone network provider Airtel. From these, data were transferred through short message service (SMS) using the Airtel network. HS without mobile network coverage continued following the same procedures as the other regions of Madagascar. Within its goal of capacity reinforcement and fight against epidemics, the “Health watch” (Veille Sanitaire) project of the Indian Ocean Commission (IOC) has ensured continuation of this reinforced IDSR in the south. In 2013, data transfer by SMS was also introduced in the two regions in the southeast. Here, out of the 258 HS, 142 (55%) had access to the Airtel mobile phone network and were included.

A summary of the IDSR approach reinforced through SMS data transfer in the south and southeast of Madagascar is provided in Appendix 1.

Before extending or adjusting the SMS data transmission reinforced IDSR strategy in southern Madagascar, we evaluated the system to determine its performance and potential ways of improvement.

Our specific objectives were to evaluate its performance using the attributes simplicity, data quality, completeness and timeliness, and to evaluate the technological aspects, including mobile phone network coverage and quality, capacity of healthcare staff to handle the mobile phones, and proportion of mobile phone losses and breakdowns.

## Methods

We adapted evaluation guidelines published by WHO, the Morbidity and Mortality Weekly Report (MMWR) and from United States Centers for Disease Control and Prevention (CDC) to our context [7–9].

### Indicators to be collected and definitions

For each of our attributes to be evaluated (simplicity, data quality, completeness and timeliness, and the technological assessment) we defined an indicator to be measured, as described in Table 1 (IDSR evaluation attributes and indicators, south and southeast of Madagascar, 2014–15). We also defined points of action that could be undertaken depending on the evaluation results (not shown).

**Table 1:** IDSR evaluation attributes and indicators, south and southeast of Madagascar, 2014–15

Attribute/ topic	Indicators	Numerator/ denominator
Simplicity		
Ease of understanding	Presence of Terms of Reference (TOR) in the HS	Number of HS agents possessing a (SIMR) TOR document/ Number of interviewed HS agents
	Proportion of HS agents capable of describing the activities linked to the surveillance (according to TOR)	Number of HS agents who could describe the activities linked to the surveillance/ Number of interviewed HS agents
Ease of execution	Proportion of HS agents who	
	• Master selected case definitions (*acute respiratory infection (ARI)*, *diarrhoea*, *malaria*, *dengue-like syndrome (DLS),* and *measles*)	• Number of HS agents who correctly cited all case definition aspects/ Number of interviewed HS agents
	• Presence of case definitions guidelines in the HS	• Number of HS agents possessing hardcopy case definitions/ Number of interviewed HS agents
	Distribution of data collection mode and kind of tools used	Number per mode or tool/ Number of modes or kind of tools used
	Time of data collection	Median and range of minutes needed each week
	Time of SMS editing	Median and range of minutes needed to write one SMS
Data quality		
Missing data	Number and proportion of SMS with ≥1 missing observation among the ten last SMS sent for *frequent diseases* (see under definitions below)/ syndrome	Number of SMS with ≥1 missing observation/ 10 last SMS sent
	Proportion of SMS with ≥1 missing observation among the ten last SMS sent for *rare diseases (see under definitions below)*/ syndrome	Number of SMS with ≥1 missing observation/ 10 last SMS sent
Erroneous data	Comparison of consultation register and sent SMS archived on the HS’s mobile phone, when this was not possible (no SMS archive), data from the consultation register was compared to the databases at district or central level	
	Proportion of erroneous observations among 10 last SMS sent	Number of erroneous observations/ Number of observations sent
	Number of erroneous observations within the 10 last SMS sent	Median and range of erroneous observations
	Number of supervision visits in 2014	Median and range of supervision visits in 2014
Completeness & Timeliness		
Routine completeness	Proportion of SMS reports received over last 4 weeks	Number of SMS received/ Number of SMS expected
	Distribution of reasons for not sending SMS reports	Number of HS agents invoking each reason/ Number of reasons (for not sending SMS) quoted
Routine timeliness	Proportion of routine SMS received in time (see under definitions below) for the last 4 weeks	Number of SMS in time per week/ Number of expected SMS
	Distribution of reasons for not sending the SMS in time over last 4 weeks	Number of HS agents invoking each reason/ Number of quoted reasons
Alert notifications	Number of HS that notified alerts	
	Number of alerts notified by HS in 2014	
	Type of notified alerts	
	Proportion of alert notifications received in time (see under definitions below) for the last 4 alerts	Number of alerts received in time / Number of alerts received
Technological evaluation		
Geographical mobile phone network coverage and coverage at/around HS with the three available providers		Verification during HS visits or during telephone interview
Sources of mobile phones used for data transfer		Number of HS by phone source/ Number all HS mobile phones
Mobile phone changes/ replacements since arrival on job		Number of mobile phone changes/ replacements
Mobile phone handling capacity by HS agents (following demonstration by evaluation team)		Number of HS agents by capacity/ Number of all interviewed HS agents
Energy sources, availability and capacity		Evaluation by the evaluators in the field during HS visits
Last problem experienced with mobile phone charging		Interview with the HS director
		Number of HS by time of last problem/ Number all HS

Definitions

Missing observations = HS failing to report disease, syndrome or event data, which in the frame of a “zero reporting” (i.e. reporting even if zero cases) system should not happen

Erroneous observations = observation transferred by SMS that did not correspond to those in the consultation register

Outliers/ outlying observations = incoherent observations identified through exploration of each variables, extreme observations were verified

Completeness = Number of received weekly SMS/ Number of weekly SMS expected

Timeliness = Number of reports received within 48 h after the week in question/ Number of expected surveillance reports

Frequent disease/syndromes examples = selected diseases/syndromes, notably diarrhoea, Acute respiratory infections (ARI), malaria, Dengue like syndrome (DLS)

Rare diseases/syndromes = selected diseases/syndromes of those for which one case is defined as an epidemic, notably measles, Acute Flaccid Paralysis (AFP), plague

The study period differed depending on the attribute and indicators evaluated but all lay within 2014. Simplicity and technological indicators were evaluated at time of visit through observation or interview. To assess data quality we considered the last ten sent SMS, for supervision visits and alerts by HS the evaluation period concerned the year 2014, while for routine and alert notification completeness and timeliness we looked at the 4 weeks or four alerts preceding our evaluation interviews respectively.

### Levels included in the evaluation and healthcare structure selection

Each of the 18 districts in the south and southeast of Madagascar has a District Health Office (DHO). Two-hundred-and-ninety-four HS participate in the reinforced IDSR system; they are divided into three types:

- BHC 1: paramedical staff covering a population of 5000–9000 inhabitants
- BHC 2: usually general practitioners and paramedical staff covering > 9000 inhabitants
- Primary Care Reference Centre (PCRC, district level)

We stratified all HS according to the possibility or not to visit them in person (parts of Madagascar are considered “red zones” that cannot be visited for security reasons). Then we stratified the 184 accessible HS according to type (BHC1/2, PCRC), and selected a random sample of 23% of the HS in each stratum, resulting in 42 HS to be visited in person (Appendix 2).

We also selected a random sample of 38 of the 110 inaccessible HS for telephone interviews.

### Data collection

Our evaluation had three components, [1] the description of the surveillance system (not presented here, but summarized in Appendix 1), [2] the evaluation of its attributes, and [3] a technological assessment. We performed field visits to a selection of HS and conducted telephone interviews with a second selection.

We trained three teams including epidemiologists and a person responsible for the technology assessment) on all aspects of the evaluation. We used two questionnaires created with Wepi (www.wepi.org) that were tested and revised before being administered during the evaluation: one for HS and one for the technological assessment. In the HS, the teams interviewed the head of the HS or the agent responsible for the IDSR activities.

The telephone interviews, using the same questionnaires, took place after the teams’ return from the field. Information that needed in visu verification (for example comparison of consultation register and sent SMS, and most of the technology evaluation) could not be collected for the HS interviewed by phone. For the HS visited in person that did not have a sent SMS archive on their phone, we compared data from the consultation register with that of the IDSR databases at district level.

### Data analysis

We verified the data collected through the questionnaires checking each individual variable for coherence, missing observations and potential mistakes, before calculating the indicators for each of the surveillance attributes. We also compared the indicator results for the different types of HS that were included in the evaluation, for example urban vs. rural, background/training of HS agent, type of HS (BHC1, BHC2, PCRC), by district, region, accessibility with the Chi-square test for homogeneity.

## Results

### Description of visited and interviewed HS

We visited 42 HS between 26 November and 12 December 2014. In January and February 2015, we interviewed 38 HS by telephone. Of the 80 included HS, 61 were BHC1 (out of all 245 BHC1, 25%), 14 BHC2 (out of all 49, 29%), and 5 PCRC (out of all 18, 28%) (Table 2).

**Table 2:** Included HS by type, Madagascar, 2014–15

Type of HS	Total HS	Number included HS	Proportion (%)
Centre de Santé de Base niveau 2 (BHC 2)	245	61	25
Centre de Santé de Base niveau 1 (BHC 1)	49	14	29
Centre Hospitalier du District (PCRC)	18	5	28
Total	312	80	26

The majority of the 80 interviewed agents were paramedical staff (66%), the rest were medical doctors (34%); this was similar for both (south and southeast) regions. The time they had been in their position at time of the interview ranged between two days and nine years, with a median of one year. Sixty five percent (53/80) had previously received a surveillance training course, 20 (24%) had on-the-job-training, and nine (11%) were instructed by their predecessor (two had a combination of these). Fifty-three agents (79%) had received the last training within the previous two years.

### Attributes evaluation: Simplicity, data quality, completeness and timeliness

All results relating to the evaluated surveillance attributes are summarized in Table 3 (Indicator results by reinforced IDSR evaluation attribute, Madagascar, 2014–15).

**Table 3:** Indicator results by reinforced IDSR evaluation attribute, Madagascar, 2014–15

Indicators per attribute	Denominator	Number	Proportion (%)
Simplicity			
TOR presence	80	15	19
TOR knowledge, description of surveillance activities (Number of correct answers/ 5 questions)			
5/5		29	36
4/5		26	33
3/5		12	15
2/5		10	13
1/5		3	4
Knowledge of selected case definitions			
Malaria		66	83
Diarrhoea		62	78
ARI		37	46
Measles		14	18
DLS		13	16
Case definitions guidelines presence		51	64
Data collection mode			
Weekly		60	75
End of each day		16	20
Other		4	5
Tools routinely used for data compilation^a^			
Data form		39	35
SMS register notebook		13	12
Dashboard		12	11
Other		48	43
Time for data compilation (minutes), median (range)	42	30	(5–180)
Time for SMS writing (minutes), median (range)		5	(1–20)
Data quality			
Missing data	80		
Number of last 10 SMS with ≥1 missing observation			
Frequent diseases			
> 4		5	6
1–3		10	12
0		38	47
No response^b^		27	34
Rare diseases			
10		12	15
0		68	85
Erroneous data			
Number of 10 last SMS with ≥1 erroneous observations	42		
0		2	5
3–5		8	19
6–8		16	38
9–10		18	43
Number of erroneous observations, median (range)		12	(0–51)
Number of supervisions in 2014, median (range)	80	2	(0–26)
Completeness & timeliness			
Completeness of HS routine data transfer over last 4 weeks (SMS number)	80 (320)	58 (232)	73
Reasons for non-completeness			
Monthly DHO meeting		4	17
Training		4	17
Illness		3	13
Lost telephone or SIM card		3	13
Telephone network problem		2	9
No telephone credit		2	9
No telephone network		2	9
End of the year workload too high		2	9
Newly recruited health agent		1	4
Timeliness of routine SMS over 4 last weeks			
4/4		34	44
3/4		11	14
2/4		11	14
1/4		6	8
0/4		15	19
Reasons for non-timeliness over last 4 weeks			
Workload too high		9	24
Telephone network problem		6	16
Training		4	11
Illness		4	11
No telephone credit		4	10
Family problem, leave, or rest after on-call duty		3	8
No telephone network		2	5
No/ lost telephone		2	5
Battery charging problem		2	5
Monthly DHO meeting		1	3
Newly recruited health agent		1	3
Number of HS that notified alerts (79 alerts in total) over last 4 weeks	80	38	48
Type of notified alerts	53		
Increase malaria cases		17	32
AFP		8	15
Dog bite		8	15
Measles suspicion		8	15
Maternal death		3	6
Chikungunya		2	4
Diarrhoea		2	4
Other		5	9
Timeliness of alert notification (4 last alerts)	38		
4/4		4	10
3/4		2	5
2/4		4	10
1/4		23	61
0/4		5	13
Technological evaluation			
Geographical mobile phone network coverage and coverage at/around HS (Fig. 2)	80		
Sources of mobile phone used for data transfer			
WHO		50	63
Non-WHO		27	34
Does not know		2	3
No mobile phone		1	1
Mobile phone changes/ replacements since job start			
Not since arrival		49	61
Once		25	31
Twice		3	4
Three times		2	3
Does not use WHO provided mobile phone		1	1
Mobile phone handling capacity by HS agents	40		
Easily		31	74
Not checked		1	2
Some difficulties		6	14
Very difficult		4	10
Problems encountered (≥1 possible)	81		
No electricity/ lack of charging possibilities		34	42
No/ broken mobile phone charger		6	7
Phone battery faulty		11	14
Other		30	37
Energy sources*	98		
Solar energy		43	49
Electricity grid		25	29
Generator		17	20
Car battery		2	2
Last mobile phone charging problem (in months)	80		
< 1		30	38
1–3		1	1
3–6		3	4
> 6		29	36
No problem		17	21

^a^more than one answer possible

^b^comparison not possible as sent SMS not archived, consultation register or databases at district level not available

#### Simplicity

Fifteen (19%) of the 80 interviewed agents had terms of reference (TOR) at their work place, and 64% of them were fully familiar with their surveillance activities. Fifty-one agents (64%) had the case definitions guidelines in their HS. The best-known case definition was that for malaria (83%), followed by those of diarrhoea (78%), acute respiratory infections (ARI) (46%) and measles (18%). The case definition of dengue-like syndrome (DLS) was known by 16% overall, by 93% (26/28) in Vatovavy Fitovinany and by 81% (13/16) Atsimo Atsinanana.

Sixty agents (75%) did a weekly compilation of data from the consultation register and 20% compiled them daily. To do this, 39 (35%) used data forms, 13 (12%) had a notebook to register sent SMS, 12 (11%) had a case compilation dashboard and 48 (43%) used other tools. For the 42 agents visited in person, these other tools were confirmed to be disposable note sheets (31%), the consultation register itself (19%) or the weekly overall surveillance report notebook (17%). Fifty-seven percent (24/42) of agents use more than one tool for IDSR data compilation. The reported median time needed to compile the IDSR data was 30 min (range 5–180), that to write and send the SMS was five minutes (range 1–20). This differed by region and by background/training of the HS agent. In the south the median weekly data compilation took 24 and in the southeast 47 min. In HS with medical doctors, 50% needed 30 min or less for data compilation, in HS with paramedical staff it was 59% (*p* = 0.469). Data compilation took longer in the southeast, where 79% needed > 30 min compared to 21% in the south (*p* < 0.001).

#### Data quality

Sixty-seven percent (28/42) of the HS visited in person did not have an archive of sent SMS on their mobile phone.

Thirty-eight of the 80 (47%) interviewed HS had no missing observations for selected frequent diseases within the last ten sent SMS and 5 (6%) had more than four SMS (out of ten) with ≥1 missing observation. For rare diseases/syndromes, 68 (85%) of HS had no missing observations.

Eighteen (43%) of the 42 HS visited in person had erroneous observations in 9–10 out of the last ten SMS that were transferred, 16 (38%) had 6–8 mistakes. Two (5%) of them had no mistakes. The mean number of erroneous observations in the last ten sent SMS was 12 (range 0–51). Eleven (65%) HS with medical agents and 22 (58%) with paramedical agents had transmitted > 10 erroneous observations among the last ten SMS sent (*p* = 0.637). By type of HS, four (10%) BHC1 had > 10 erroneous observations, 27 (40%) BHC2, and 2 (5%) PCRC (*p* = 0.179). There were more erroneous data transmitted from the southeast (67%) compared to the south (33%) region (*p* = 0.004).

The median number of supervisions the 80 interviewed HS had in 2014 was 2 (range 0–26), there was no difference between the south and southeast.

#### Completeness and timeliness

In the four weeks preceding our interviews, the routine data transfer completeness was 73% (232/320). Main reasons for non-completeness cited by the agents were the monthly DHO meeting (17%), training (17%), illness (13%) or lost telephone and/or subscriber identification module (SIM) card (13%).

The overall timeliness of routine reporting was 43% (136/320). Forty-four percent (34/77) of HS sent all four of the SMS of the previous four weeks in time, and 19% did not send any of the four SMS in time. The reasons for non-timeliness were high workload (24%), telephone network problem (16%), training or illness (11% each) and illness (10%).

Almost half (38/80, or 48%) of the HS issued an alert over the four weeks before our interviews, and in total 79 alerts were notified. Among the 53 alerts for which information was available, an increase in malaria cases was the most frequently notified event (32%), followed by acute flaccid paralysis (AFP) cases, dog bites, and measles suspicion (15% each). Out of the 38 HS that notified an alert, 10% (4/38) notified all last four events in time.

Thirty-five (70%) of the HS with paramedical agents had transferred data in time, for those with medical agents 21 (78%) did timely data transfer (*p* = 0.467). Routine data transfer was timely by 9 (69%) BHC1, 44 (73%) BHC2 and 3 (75%) PCRC (*p* = 0.950) respectively.

### Technological evaluation

**Fig. 2:**
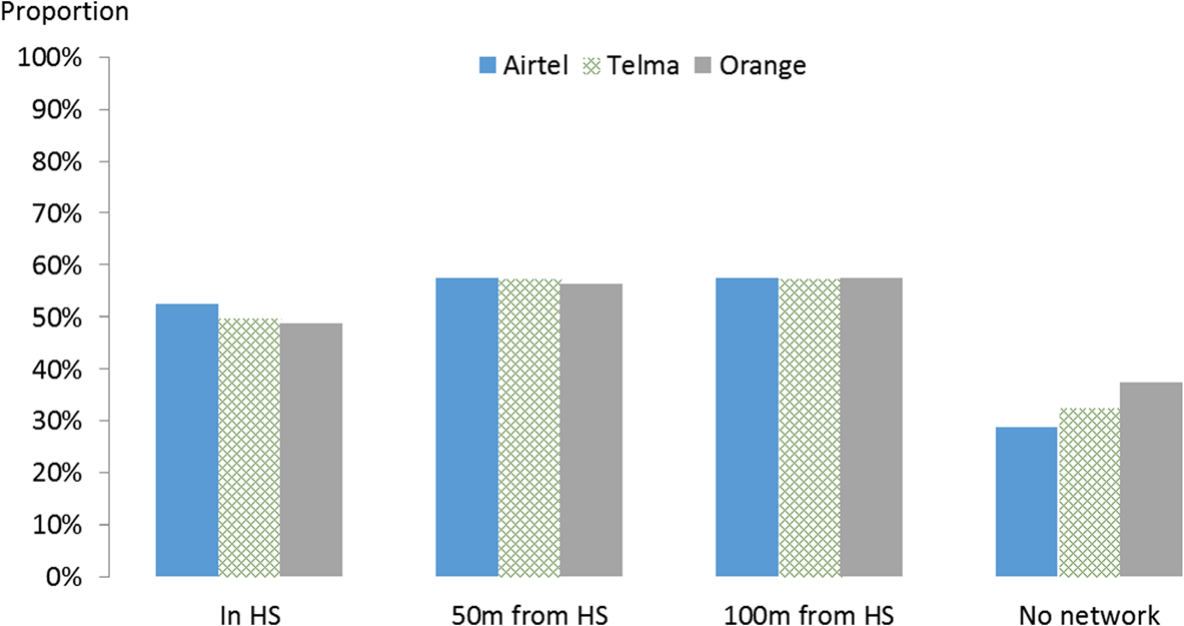
Mobile phone network coverage at/around HS (*N* = 80), south and south-east, Madagascar, 2014. Bar chart illustrating the coverage by each of the three available mobile phone network providers within the HS and at 50 and 100 m distance

The geographical mobile phone network coverage by each of the three available providers (Airtel, Telma, Orange) detected during evaluation of the 80 HS is illustrated in Fig. 2 (Mobile phone network coverage at/around HS (*N* = 80), south and south-east, Madagascar, 2014). Between 39 (49%) and 42 (53%) HS had mobile phone coverage within their structure depending on the provider; this proportion slightly increased when the area around the HS was explored for coverage (up to 58%). Coverage was slightly higher with Airtel, the currently used IDSR network provider, compared to the other two mobile phone companies’ networks. However, 23 (29%) of the evaluated HS were not covered by the Airtel network at all.

Sixty-three percent (50/80) of the mobile phones used by the 80 interviewed HS agents during our evaluation came from WHO, who originally provided them for the reinforced IDSR. Two HS (3%) did not know the phone’s origin and one did not have a phone. For 49 (61%) HS agents, there had been no mobile phone changes or replacements since their start on the position, 31% had had one replacement, 7% more than one. Nearly three quarters (31/42) could easily handle a mobile phone under observation, whereas 24% had some or greater difficulty with this.

Eighty-one problems with mobile phones were reported, with 8% (6/80) of HS mentioning more than one. Of these, 63% (51/81) were related to diverse aspects of mobile phone charging: electricity cuts or problems (42%), lack of or broken charger (7%) or faulty phone battery (14%).

Some HS used more than one energy source, the most frequently used one was solar energy (43/87, or 49%), followed by grid electricity (29%), electric generator (20%) and car battery (2%). Seventeen (21%) of the HS reported not having had a problem to charge the mobile phone, for 36% the last problem was more than 6 months ago, while 38% reported having had problem with this within the last month (Table 3).

## Discussion

The evaluation of the reinforced IDSR strategy in the south and southeast of Madagascar allowed us to assess the system’s functioning and identify challenges to be addressed before introduction of mobile health data collection in further districts in Madagascar.

### Simplicity

The evaluation of the IDSR activities at HS level raised a number of issues regarding knowledge of terms of reference, surveillance procedures, and case definitions. These could be due to the lack of guidelines and documents, for example TOR and case definitions, in the HS. In some HS the turnover of agents is high, and not all receive IDSR training.

Knowledge of case definitions was better for frequent diseases/syndromes like malaria and diarrhoea. In a similar way, HS agents in regions where DLS is prevalent were more familiar with the clinical case definition. Similar issues related to lack of guidelines, training, and supervision have been identified in other countries [10, 11].

Data collection, compilation and writing (in SMS form) was not straightforward and time-consuming depending on peoples’ familiarity with the procedures and dexterity with mobile phones. More than half of the agents used more than one tool for the weekly data compilation. On average, the preparation and sending of the data each week takes the interviewed HS agents 35 min, even if this differed by region and took agents in the southeast longer. This and the larger proportion of erroneous data from the southeast might be due to its more recent inclusion. We compared results according to data collection method for indicators to explore potential bias (ease of execution of surveillance activities). There was no evidence for a difference between results from HS visited in person vs. those interviewed by telephone.

In order for disease surveillance systems to be effective, it is crucial they are simple to understand and perform [2]. In principle, electronic data transfer is supposed to improve data quality [12], but we found many problems that might be related to the case ascertainment (case definition knowledge) and data compilation steps.

Regular training and supervision would allow improving knowledge of IDSR methods and activities, and standardisation of data compilation in a simple manner across HS would be a solution to increase HS agents’ mastering of IDSR activities [13].

### Data quality

The quality of the data collected within the reinforced IDSR surveillance shows there is room for improvement. Albeit better for rare diseases, half of the HS transferred data with missing observations within the ten-week period assessed during the evaluation. Another issue undermining the reliability of the surveillance results was the amount of erroneous data transferred by the HS. Only a small proportion of transferred SMS had no mistakes. This does not seem to be related to the type of HS or training level of the responsible agent, even though the numbers might have been too small to detect a difference. The data quality problems are also related to points discussed under simplicity, notably lack of guidelines and training [10, 13]. They could further be explained by the small number of supervision visits that took place in the year preceding the evaluation, as well as the already mentioned high agent turnover. Colleagues in Madagascar recently evaluated the national sentinel influenza surveillance system that includes 34 HS and is also based on SMS transmission of aggregated data. It performed well regarding the quality of the data collected, but pointed out a need for improving staff training [14].

### Completeness & timeliness

Completeness and timeliness were too low to respond to the surveillance objectives. This concerns in particular the detection of unexpected health events. HS agents reported a high workload and technical problems as the main challenges they face with regards to routine data transfer. We could not assess completeness of alert notification, since there was no reliable system or register to which the transferred data could have been compared. Timeliness of alert notification was poor, and early detection of disease outbreaks in the evaluated areas is not ensured.

Simplification of data compilation and transfer, as well as ensuring working technologies (chargeable mobile phone and functioning android application), could help with improving these two attributes [15]. Closer supervision and support of HS IDSR activities could also help with improving these attributes’ outcome, but these would come at a price and cost-effectiveness might need to be evaluated [16].

### Technology evaluation

Not all aspects of the evaluation could be verified for those HS that were interviewed by telephone.

While more industrialised countries are most implementing electronic medical records and/or data transfer, this remains a financial and logistical challenge in many African states [17]. The increase in new technologies that can support epidemiological surveillance has made a positive difference in performance and data quality [15, 18].

Finally, we are convinced that regular results’ feedback to those who provide the surveillance data could help raising interest, dedication and motivation of HS agents responsible of IDSR. This could have a positive impact on several of the surveillance attributes we evaluated, notably simplicity, data quality, completeness and timeliness [13].

We believe that the reinforced IDSR surveillance should not be limited to one mobile network provider but that the choice of these should be based on network availability at each HS, to increase realistic coverage.

### Recommendations

Following our evaluation, we recommended to the IDSR collaborators and the Ministry of Health to:

- Revisit choice of HS included in the system according to mobile phone network coverage
- Produce and distribute simple, understandable TOR and case definition guidelines that can be displayed within the HS
- Reinforce capacities of the persons involved in surveillance activities through supervisory training
- Improve data collection, compilation and transfer by rendering it electronic
- Add other mobile phone network providers to increase coverage of HS in the regions

## Conclusion

Early detection of unexpected health events is crucial to minimise the impact of epidemics [10, 19]. The IDSR approach is suitable for this, but it needs to be adapted to each specific context. Simple procedures, physical presence of guidelines and support material, as well as training and supervision are key to making it a success.

In Madagascar’s southern regions, SMS transfer has improved IDSR data completeness, but timeliness and data quality remain a problem. Healthcare staff needs training on IDSR guidelines and case definitions. Since May 2016, data are collected and managed electronically in several pilot districts in Madagascar to reduce errors and improve the system’s performance. Since April 2017, a weekly surveillance bulletin is circulated to central level, regional and district health offices. We hope this bulletin will also be accessible to the data providers, to show them the use and benefit of their IDSR-related work activities.

## Figures and Tables

**Fig. 3:**
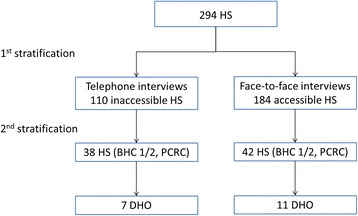
Selection of HS included in the reinforced IDSR strategy evaluation, Madagascar. Flowchart describing the selection of HS included in the evaluation following the stratification criteria described in the methods section

## Appendix

### Appendix 1

**Table 4:** Description of the SMS-reinforced IDSR strategy. Table describing characteristics of the reinforced IDSR strategy in southern Madagascar including objectives, indicators, data source, collection, transfer and use

Characteristic	Description
PH importance of diseases/ syndromes under surveillance	Diseases under surveillance constitute the biggest part of basic healthcare consultations such as Acute Respiratory Infections (ARI), malaria, diarrhoea
Available interventions	23/35 of the diseases and syndromes under surveillance have a defined response foreseen in the national health action plan
IDSR objectives	Follow the trend of endemic and/or epidemic diseases and syndromes
	Detect cases of highly epidemic diseases or diseases subject to elimination or eradication programmes as well as unexpected events in a timely manner
	Provide IHR data to the WHO
Performance indicators used	Weekly number of cases and deaths by disease, syndrome or event, and by HS
	Proportional morbidity: Number of consultations by disease, syndrome or event/ Number of total consultations
	Completeness: Number of reports received/Number of expected surveillance reports
	Timeliness: Number of reports received within 48 h after the week in question/Number of expected surveillance reports
Information collected	Number of cases and deaths for three groups of diseases/syndromes/ events
	• Endemic and potential epidemic diseases: Acute Respiratory Infections (ARI), diarrhoeal diseases, malnutrition, malaria, tuberculosis, Human Immunodeficiency Virus (HIV), Sexually Transmitted Infections (STI), maternal deaths
	• Highly epidemic diseases: cholera, bacterial dysentery, meningitis, plague, yellow fever, viral haemorrhagic fever, chikungunya, dengue-like syndrome (DLS), rabies, foodborne outbreaks, severe acute respiratory syndrome (SARS), avian influenza, Rift Valley fever, chickenpox, West Nile virus
	• Diseases subject to eradication or elimination programmes: poliomyelitis & acute flaccid paralysis (AFP), leprosy, measles, neonatal tetanus, filariasis, malaria
Data source	HS patient consultation register
Data collection, entry and transfer	Compilation of weekly number of total consultations, and cases and deaths per disease/ syndrome or event (including zero reporting) before sending them per SMS to the DHO. At the DHO, the surveillance focal point enters the data into an Excel spreadsheet.
Database set-up	One observation (line) per week and per district
Data analysis and thresholds	Weekly analyses based on defined thresholds, for example:
	• Disease for which one case = epidemic, such as meningitis, acute flaccid paralysis, neonatal tetanus, measles, SARI, avian influenza, cholera, plague, haemorrhagic fever, human rabies
	• Malaria: doubling of cases over three consecutive weeks
	• Brutal increase in comparison with other diseases/ syndromes
	• Completeness and timeliness of data transfer
Communication	No routine communication of analysis results to stakeholders (2016)
Use of data and analyses results	Weekly monitoring of performance indicators (completeness, timeliness), investigation of and response to potential identified or notified signals

## Abbreviations

AFP: Acute flaccid paralysis
ARI: Acute respiratory infection
BCH: Basic Healthcare Centre
CERF: Central emergency response fund
DHO: District Health Office
DLS: Dengue-like syndrome
DVSSE: Direction de Veille Sanitaire et Surveillance Epidémiologique (Epidemiological Surveillance Department)
HS: Health structure (any level)
IDSR: Integrated disease surveillance and response strategy
IHR: International health regulations
IOC: Indian Ocean Commission
PCRC: Primary Care Reference Centre
PH: Public health
SARI: Severe acute respiratory syndrome
SMS: Short message service
TOR: Terms of reference
WHO (AFRO): World Health Organization (Regional Office for Africa)
